# Comparative analysis of detoxification-related gene superfamilies across five hemipteran species

**DOI:** 10.1186/s12864-022-08974-y

**Published:** 2022-11-17

**Authors:** Mariano Volonté, Lucila Traverso, Jose Manuel Latorre Estivalis, Francisca Cunha Almeida, Sheila Ons

**Affiliations:** 1 Laboratorio de Neurobiología de Insectos (LNI), Centro Regional de Estudios Genómicos, Facultad de Ciencias Exactas, Universidad Nacional de La Plata, CENEXA, CONICET, La Plata, Buenos Aires Argentina; 2 Laboratorio de Insectos Sociales, Instituto de Fisiología, Biología Molecular y Neurociencias, Universidad de Buenos Aires – CONICET, Ciudad Autónoma de Buenos Aires, Argentina; 3 Grupo de Investigación en Filogeografía y Filogenias Moleculares, Departamento de Ecología, Genética y Evolución, Facultad de Ciencias Exactas y Naturales, Universidad de Buenos Aires, Ciudad Autónoma de Buenos Aires, Argentina

**Keywords:** Glutathione transferases, Cytochromes P450, Carboxyl/cholinesterases, Insects, Pentatomid, Triatomine, Heteroptera

## Abstract

**Background:**

Hemiptera is one of the most speciose orders of insects, and the most speciose considering Hemimetabola. Through their evolutive history, hemipterans with different feeding habits have adapted to deal with different chemical challenges. Three major gene families are involved in xenobiotic detoxification in insects: the cytochromes P450 (CYPs), carboxyl/cholinesterases (CCEs), and glutathione transferases (GSTs). Here we perform a comparative analysis on the complement of these gene superfamilies across five hemipteran species; four heteropterans (the pentatomid plant feeders *Nezara viridula* and *Halyomorpha halys*; the hematophagous *Cimex lectularius*, Cimicidae, and *Rhodnius prolixus,* Reduviidae), and one Auchenorrhyncha plant feeder (*Nilaparvata lugens*).

**Results:**

Our results point to an expansion of several enzyme families associated with xenobiotic detoxification in heteropterans with respect to other species and the existence of a dynamic evolution pattern including CYP3 clan, hormone and pheromone processing class in the CCE superfamily, and sigma class in GST superfamily. Other detoxification-related families are reduced in the hemipteran species analyzed here: reduction or even absence of epsilon class and reduced delta class in GST superfamily; absence of mitochondrial CYP12 family; absence of CYP9 family in CYP3 clan; and reduction or even absence of some dietary/detoxification groups of CCEs. Interestingly, the most polyphagous species analyzed here (*H. halys*) is also the one that presents the largest repertoire of detoxification enzymes. Gene cluster analysis suggests that this could be due to gene duplication events.

**Conclusions:**

The evolutionary analysis performed here reveals characteristics that are both common and particular for heteropterans. The composition and organization of detoxification-related gene families could shed light on evolutionary forces that shaped their divergence. These families are important for both the detoxification of diet products and for conferring tolerance or resistance to synthetic insecticides. Furthermore, we present the first comprehensive analysis of detoxification gene superfamilies in *N. viridula*, an understudied species in spite of its economic relevance as a crop pest. The information obtained is of interest for basic insect science as well as for the control of harmful species and the management of insecticide resistance.

**Supplementary Information:**

The online version contains supplementary material available at 10.1186/s12864-022-08974-y.

## Background

Hemiptera is one of the most speciose orders of insects, and the most speciose considering Hemimetabola; it comprises more than 50,000 species. The suborder Heteroptera (true bugs) includes species adapted to a diversity of ecological niches and lifestyles. Phylogenomic analysis and fossil records suggest that the origin of the Heteroptera suborder is coincident with a shift from herbivory to predation [1], whereas in Pentatomorpha and Cimicomorpha, two lineages within Heteroptera, a shift back to herbivory occurred. Within Cimicomorpha, hematophagy emerged independently in the Triatominae subfamily (kissing bugs) and the Cimicidae family (bed bugs). Dissimilar diets present different challenges for these phylogenetically related heteropterans. Through their evolutive history, hemipterans with different feeding habits have adapted their detoxification physiology to deal with different chemicals. Whereas phytophagous insects are exposed to toxins, repellents or anti-digestive compounds from plants [2], hematophagous insects ingest toxic amounts of heme, iron, and amino acids in every meal [3].

Three major gene families are involved in xenobiotic detoxification in insects: the cytochromes P450 (CYPs), carboxyl/cholinesterases (CCEs), and glutathione transferases (GSTs) [4]. It has been largely demonstrated that the ability of herbivorous insects to detoxify plant allelochemicals affects their capability to utilize different plants as potential hosts [5]. In fact, a positive correlation between the number of detoxification genes in a particular species and the complexity of its food sources has been reported [5]. On the other hand, a reduced complement of detoxification enzymes in species such as the honey bee *Apis mellifera* [6] or the human head louse *Pediculus humanus* [7] was related to their specialized diet. Likewise, generalist hymenopterans seem to present a higher number of GSTs, CCEs, and CYPs when compared to specialist species of the same order [8]. However, a recent comparative analysis on the Aphidinae subfamily (Hemiptera: Aphididae) indicates that there is no correlation between the host range of a species and the number of detoxification-related genes encoded in its genome, suggesting that this correlation could not be generalized for hemipteran species [9].

It is generally accepted that expansions or contractions of detoxification-related gene families throughout evolution are associated with functional adaptations to the diet range and other characteristics from the environment [8]. The evolution of gene families is determined by a dynamic process of gene duplication and loss (the gene birth-and-death model), which is mediated by mutation genetic drift, natural selection, and chromosomal rearrangements [10]. Paralogue gene proliferations in genomes (or “gene blooms”) originate from gene duplications, causing significant expansions in particular subfamilies [11]. Newly duplicated genes can be fixed if they are adaptive, or lost through deletions or deleterious mutations that turn them into pseudogenes. Hence, the comparative analysis of insect detoxification-related complement could indicate those groups of genes involved in the adaptation of the species to particular ecological niches.

In previous work, we found that triatomines, which are obligate blood-feeders in their complete life cycle, present reductions (or even absence) of some families of GSTs, CCEs, and CYPs, whereas others are expanded [4]. To our knowledge, a comprehensive analysis comparing xenobiotic detoxification-related genes in hemipterans with different feeding habits has not been reported to date; hence, some of the particularities found in triatomines could be common to Heteroptera or Hemiptera. Even though a previous report compared the CYP, CCE, and GST superfamilies in 160 insect species [5] providing interesting conclusions regarding adaptations to different diets, it did not include any species of the Hemiptera order. Hence, the information on this group, relevant both in terms of number of species and its economic/sanitary impact, is still scarce. Furthermore, an evolutionary analysis identifying differences in CYP, GST, and CCE complements between sap- and blood-sucking heteropterans could reveal molecular adaptations to either hematophagy or plant feeding. A comparative analysis of gene expansions/reductions and selective pressure between blood- and sap-sucking species could reveal common characteristics within the heteropterans, and also differences that could shed light on the evolutive forces exerted on different genes and gene families.

The stink bug *Nezara viridula* (Hemiptera: Pentatomidae) is an important crop pest that generates a severe economic impact, particularly in soybean [12]. In spite of its economic relevance from North to South America, the genome of *N. viridula* has not been published to date, although a high-quality and complete transcriptome is available [12]. *Halyomorpha halys* (Hemiptera: Pentatomidae), also phytophagous, has exceptionally high levels of polyphagy*,* which makes it an efficient invasive plague that expanded from Asia to North and South America, Europe and Australia [13]. *Nilaparvata lugens* (Hemiptera: Delphacidae) is a planthopper that is a monophagous herbivore of rice, posing a serious threat to production [14]. The bed bug *Cimex lectularius* (Hemiptera: Cimicidae) and the kissing bug *Rhodnius prolixus* (Hemiptera: Reduviidae) are obligate hematophagous during their entire life cycle, and are species of sanitary relevance as human ectoparasites. Furthermore, *R. prolixus* is a vector of *Trypanosoma cruzi*, the causative agent of Chagas disease, a neglected life-threatening disease that affects millions of people all over the world (https://www.who.int/news-room/fact-sheets/detail/chagas-disease-(american-trypanosomiasis).

**Fig. 1:**
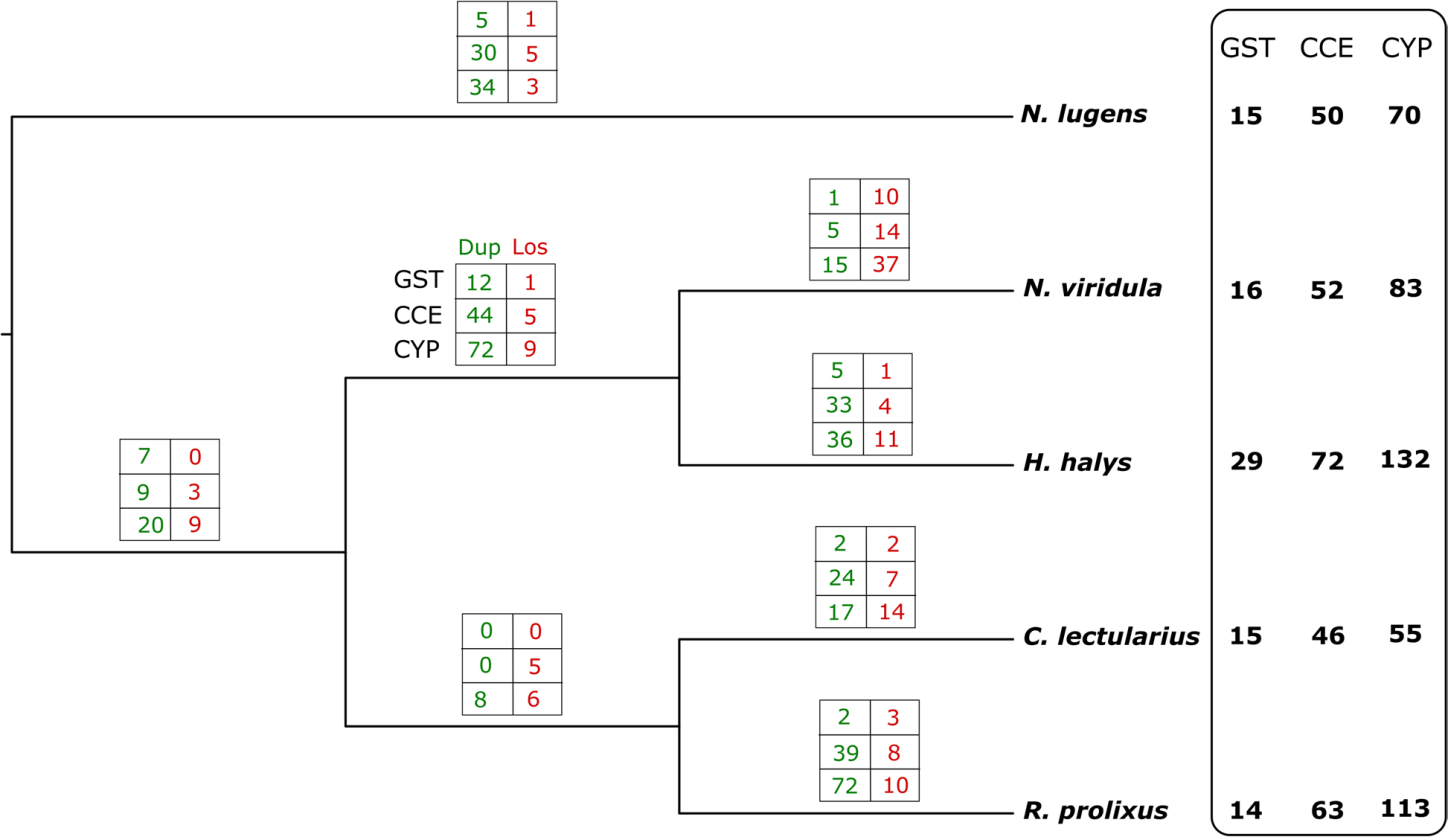
Species tree showing the number of gene gains (green) and losses (red) in each branch for GST, CCE, and CYP superfamilies

Here, we perform a comparative analysis on the complement of GST, CCE, and CYP gene superfamilies across four heteropterans (*N. viridula*, *H. halys*, *C. lectularius,* and *R. prolixus*), and one Auchenorrhyncha (*N. lugens*) (Fig. 1). The results of this analysis revealed particularities in the detoxification-related complement of Heteroptera with respect to other insect species, and pointed to gene families that could maintain a fast evolution rate.

## Results and discussion

CYP, CCE, and GST repertoires were compared across four heteropterans (*N. viridula*, *H. halys*, *R. prolixus,* and *C. lectularius*) and one Auchenorrhyncha (*N. lugens*). The latter was included in the analysis as a hemipteran non-Heteroptera outgroup. Considering the sum of members of all gene superfamilies under analysis, 151 detoxification-related genes in *N. viridula*, 116 in *C. lectularius*, 190 in *R. prolixus,* 233 in *H. halys*, and 135 in *N. lugens* were identified (Table 1). CYP superfamily was the largest in terms of the number of genes in all species, followed by CCEs, and GST was the least represented (Table 1). This trend was also observed in other genomes such as those from several Aphidinae species [9] and *Anopheles gambiae* [15] but not in other insect species; for instance, *Drosophila melanogaster* possesses more GST than CCE genes [4].

**Table 1:** Gene number of detoxification-related superfamilies and their respective families/classes per species^a^

Superfamily	Family/Clan/Class		***R. prolixus***	***C. lectularius***	***H. halys***	***N. viridula***	***N. lugens***
**CYPs**	**Mitochondrial clan**	Number	7	7	8	6	11
		Families	CYP301, 302, 314, 315, 394, 404	CYP301, 302, 314, 315, 394, 404	CYP301, 302, 314, 315, 3221	CYP301, 302, 314, 315, 3221	CYP301, 302, 314, 315, 353, 404, 419
	**CYP2 clan**	Number	6	6	6	4	9
		Families	CYP15, 18, 303, 306, 307	CYP15, 18, 303, 305, 306, 307	CYP15, 18, 303, 305, 306, 307	CYP15, 305, 306, 307	CYP15, 18, 303, 304, 305, 306, 307
	**CYP3 clan**	Number	53	30	75	41	16
		Families	CYP6, 395, 3084-3092, 3096	CYP6, 395, 396, 397, 398, 399, 400, 3087, 3089, 3090, 3091	CYP6, 395, 3090, 3092, 3226, 3227, 3228, 3229, 3230, 3231	CYP6, 395, 3090, 3092, 3225, 3226, 3227, 3228, 3229, 3230, 3231	CYP6, 408, 427, 3115
	**CYP4 clan**	Number	47	12	43	32	34
		Families	CYP4, 3093	CYP4	CYP4, 3222, 3223, 3224	CYP4, 3223, 3224	CYP4, 380, 417, 425, 426, 439
	**Total number of CYP superfamily**		**113**	**55**	**132**	**83**	**70**
**CCEs**	**Hormone and pheromone processing class**	Number	43	30	57	45	25
		Families	B-Esterases	B-Esterases, Uncharacterized	B-Esterases	B-Esterases	B-Esterases, Uncharacterized
	**Dietary class**	Number	0	0	0	0	5
		Families					A- Esterases
	**Neurodevelopmental class**	Number	20	16	15	7	20
		Families	Neuroligin, I-Class, Neurotactin, Gliotactin, Glutactin, Acetylcholinesterase	Neuroligin, I-Class, Neurotactin, Gliotactin, Glutactin, Acetylcholinesterase	Neuroligin, I-Class, Neurotactin, Gliotactin, Glutactin, Acetylcholinesterase	Neuroligin, Gliotactin, Glutactin, Acetylcholinesterase	Neuroligin, I-Class, Neurotactin, Gliotactin, Glutactin, Acetylcholinesterase
	**Total number of CCE superfamily**		**63**	**46**	**72**	**52**	**50**
**GSTs**	**Delta class**	Number	1	2	2	0	2
	**Epsilon class**		0	0	0	0	4
	**Omega class**		1	1	3	2	1
	**Sigma class**		7	6	17	9	4
	**Theta class**		3	2	3	3	1
	**Zeta class**		1	1	1	1	1
	**Microsomal class**		1	3	3	1	2
	**Total number of GST superfamily**		**14**	**15**	**29**	**16**	**15**
**Total number of detoxification-related enzymes**			**190**	**116**	**233**	**151**	**135**

^a^Numbers from *C. lectularius*, *H. halys*, *R. prolixus* and *N. lugens* genomes and *N. viridula* transcriptome

*Halyomorpha halys* had the largest repertoire in all superfamilies: 132 genes in CYP, 72 in CCE, and 29 in GST. By comparison, the *C. lectularius* repertoire was the smallest with 55 CYPs, 46 CCEs, and 15 GSTs (Table 1). The CYP and CCE superfamilies of *R. prolixus* had 113 and 63 members, respectively, followed by *N. viridula* with 83 and 52, respectively (Table 1). The GST superfamily size was more homogeneous across species with 16 genes in *N. viridula,* 15 in *C. lectularius* and *N. lugens,* 14 in *R. prolixus*, although *H. halys* diverged from the trend, with 29 genes (Table 1).

When the sum of CYPs, CCEs, and GSTs was compared to the complete gene sets in the genomes, significant expansions were detected in *H. halys* and *R. prolixus* but not in *N. lugens* and *C. lectularius* (Table A in Supplementary information 1). This was supported by different numbers of gene duplications and losses (Fig. 1), *H. halys* and *R. prolixus* being the species with the largest number of duplication events detected in the gene families studied herein.

### Cytochromes P450

CYPs are involved in the degradation of xenobiotics, from both diet and the environment, being an important factor in the response of insects to chemical stress [16]. They also participate in key metabolic processes, such as the degradation of pheromones or the biosynthesis of signaling molecules. CYP genes are divided into 4 large clans (Mitochondrial, CYP2, CYP3 and CYP4), which in turn are subdivided into families and subfamilies [17].

**Fig. 2:**
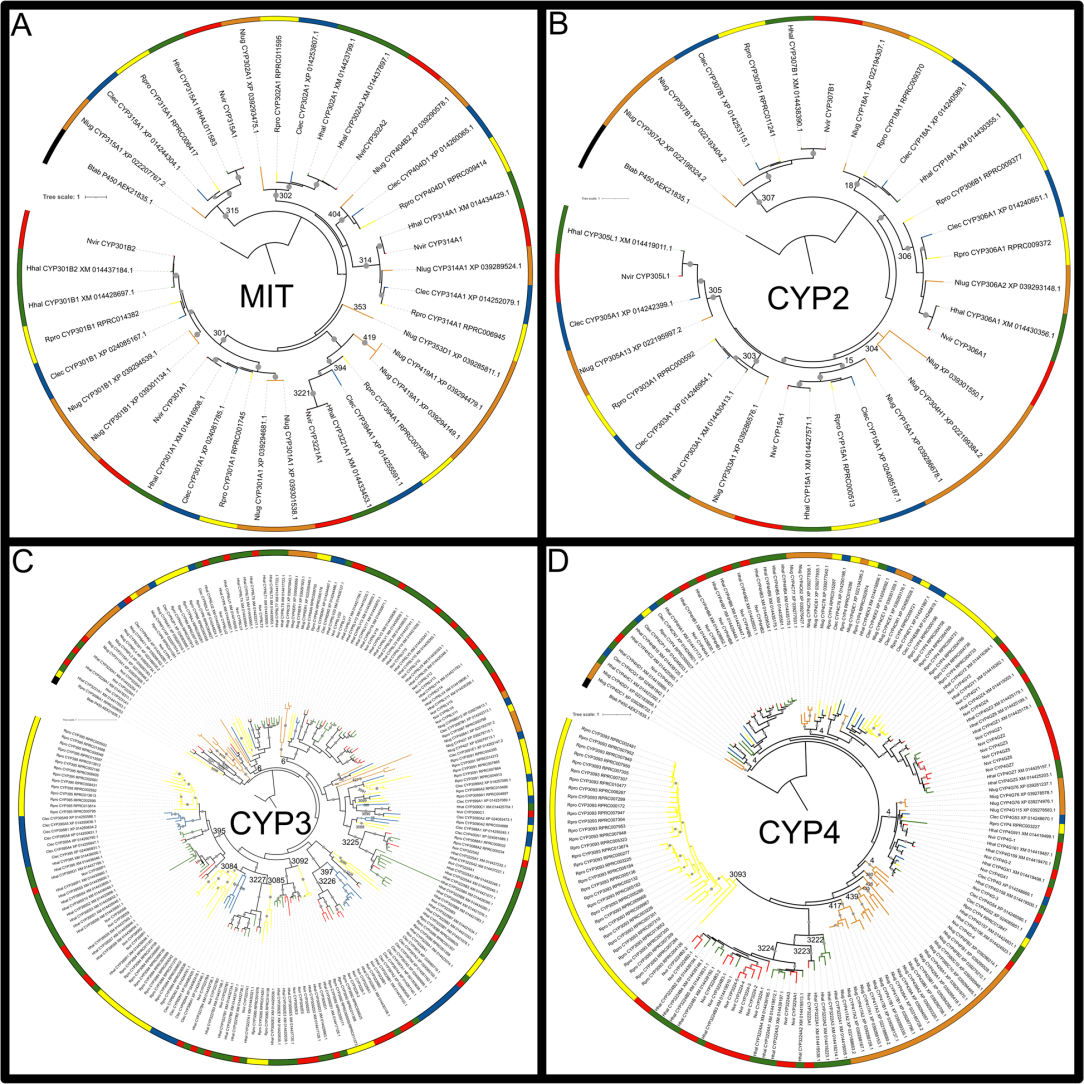
Phylogeny of CYP superfamily from *N. viridula* (Red: Nvir), *R. prolixus* (Yellow: Rpro), *H. halys* (Green: Hhal), *C. lectularius* (Blue: Clec), and *N. lugens* (Orange: Nlug). **A** Mitochondrial clan. **B** CYP2 clan. **C** CYP3 clan. **D** CYP4 clan. A CYP gene from *Bemisia tabaci* was used as an outgroup (AEK21835.1 - NCBI), and the tree was rooted on this sequence. Branch support values > 80 are marked to scale with a gray circle

The CYP complements of the *N. lugens* and *C. lectularius* genomes were significantly smaller when compared to the CYP complements of other heteropteran genomes analyzed herein. Furthermore, when comparing the amount of CYP genes within the complete detoxification repertoire, the number found in *R. prolixus* was significantly greater than in *C. lectularius* (Table A in Supplementary information 1). Our phylogenetic analysis allowed the classification of CYP superfamily members into clans and families (Fig. 2), which presented different evolutionary dynamics (Table 2).

**Table 2:** Analysis of gene gain and loss by family in the analyzed species^a^

Family	CYPMIT	CYP2	CYP3	CYP4	GST	CCE	Total
**Duplications**							
**Clec**	0	0	17	0	2	24	43
**Rpro**	0	0	34	38	2	39	113
**(Clec + Rpro)**	0	0	5	3	0	0	8
**Hhal**	0	0	25	11	5	33	74
**Nvir**	0	0	6	9	1	5	21
**(Hhal + Nvir)**	2	0	43	27	12	44	128
**(Clec + Rpro) + (Hhal + Nvir)**	0	0	15	5	7	9	36
**Nlug**	3	1	9	21	5	30	33
**Losses**							
**Clec**	1	1	10	2	2	7	23
**Rpro**	0	1	4	5	3	8	21
**(Clec + Rpro)**	0	0	3	3	0	5	11
**Hhal**	0	0	6	5	1	4	16
**Nvir**	2	2	22	11	10	14	61
**(Hhal + Nvir)**	1	1	7	0	1	5	15
**(Clec + Rpro) + (Hhal + Nvir)**	1	2	1	5	0	3	12
**Nlug**	0	1	1	1	1	5	9
**Total**	10	9	208	146	52	235	624

^a^Numbers belonging to phylogenetic analysis of *Nilaparvata lugens* (Nlug), *Cimex lectularius* (Clec), *Halyomorpha halys* (Hhal), *Rhodnius prolixus* (Rpro) genomes and *Nezara viridula* (Nvir) transcriptome

#### Mitochondrial clan

The number of mitochondrial clan genes was similar among the analyzed species (8 in *H. halys*, 7 in *C. lectularius* and *R. prolixus*, 6 in *N. viridula*, and 11 in *N. lugens,* Table 1). These numbers reflect stability in the evolution of the clan, with few events of gene duplications and losses (Table 2). Many genes of this clan have essential roles in the life cycle of insects, such as the *Halloween* genes that are involved in the synthesis of ecdysteroids [17]. *Halloween* genes belong to the 315 (*sad*), 302 (*disembodied*; *dib*), and 314 (*shadow*; *shd*) families. All of them were represented by 1 gene per species in the hemipteran databases, with the exception of two CYP302 genes in *H. halys* (Fig. 2A). The CYP394 family of the mitochondrial clan had 1 representative in *R. prolixus* and *C. lectularius*, whereas CYP3221 had 1 representative in each one of the pentatomids. *N. lugens* had no representatives of the latter two families, but it had 2 genes classified as CYP419, which is phylogenetically related. Moreover, *N. lugens* genome encodes one gene belonging to CYP353 family, which is absent in the heteropteran species analyzed in this study (Fig. 2A). Interestingly, CYP404 was represented by one gene in *N. lugens*, *C. lectularius,* and *R. prolixus*, but was absent in the pentatomids. The largest mitochondrial CYP family in the databases analyzed here was CYP301, with 4 genes detected in *N. lugens*, 3 in *H. halys*, and 2 in *C. lectularius, R. prolixus,* and *N. viridula* (Fig. 2A). Coincident with previous observations in triatomines, none of the sequences identified in the hemipterans analyzed herein was phylogenetically related to the CYP12 family that has been associated with insecticide resistance (Supplementary Fig. 1A) [17].

#### CYP2 clan

Like the Mitochondrial clan, CYP2 contains *Halloween* genes (e.g., CYP306 and CYP307 families, named *phantom* and *spook,* respectively) [17] (Fig. 2B). Nine CYP2 genes were detected in *N. lugens*, 6 in *R. prolixus, C. lectularius* and *H. halys*, and 4 in *N. viridula* (Table 1, Fig. 2B). The evolutionary dynamics of this clan was similar to that of the Mitochondrial clan, but with more gene loss events and one gene duplication in *N. lugens* (Table 2). A single member of the CYP305 and CYP307 families was detected in each species database, with the exception of two CYP307 genes in *N. lugens*, and no CYP305 genes in *R. prolixus*. For CYP15 family, one member was detected in each database. Two genes were classified as CYP306 family in *R. prolixus,* whereas 1 member was found in each of the other species under analysis. CYP304 family, previously reported in *D. melanogaster* and other insects, was represented by 1 gene in *N. lugens*, but not in the other species analyzed, suggesting a loss of this family in Heteroptera. In addition, a CYP2 gene from *N. lugens* (XP_039301550.1) could not be classified into any family. Finally, only one sequence belonging to each of the CYP18 and CYP303 families was identified in the 4 genomes analyzed, but not in the *N. viridula* transcriptome (Fig. 2B). In previous work, CYP303 and CYP18 subfamilies were not detected in transcriptomes of the kissing bugs *Triatoma dimidiata* and *Triatoma infestans* [4], which could suggest that these families are low or conditionally expressed in heteropterans, hindering their identification even in complete transcriptomic databases.

#### CYP3 clan

CYP3 and CYP4 clans were always larger than the Mitochondrial and CYP2 clans in the insect genomes [17]. These clans have proliferated as a result of gene duplication events, which allowed their diversification and neo functionalization [11]. Similar results were observed in the hemipteran species analyzed herein. The CYP3 clan has a very dynamic evolution and a significant expansion trend with 2.8-fold more gene duplications than losses (Table 2). Within the CYP3 clan, there are multiple xenobiotic-metabolizing families involved in phytochemical detoxification and insecticide resistance, mainly represented by CYP6 and CYP9 families [16].

The number of sequences encoding CYP3 enzymes was 53 in *R. prolixus*, 30 in *C. lectularius*, 75 in *H. halys,* and 41 in *N. viridula* (Table 1; Fig. 2C). The CYP3 complement from *N. lugens* was significantly reduced (16 genes) compared to its counterpart in heteropterans (Table 1, Fig. 2C). Accordingly, many gene duplications were observed in all phylogenetic branches leading to Heteroptera (15). Additionally, *R. prolixus* had 34 duplications and *C. lectularius* had 17, which explains most of the difference between these two hematophagous species. A large number of duplications was observed in the ancestor of *N. viridula* and *H. halys* (43), and again in the lineage of *H. halys* (Fig. 1, Table 2). The discrepancy between the two pentatomids could be related to differences in the breadth of their diets, although the much lower number of genes and duplications, and more gene losses detected in *N. viridula* could reflect incompleteness of its transcriptome either as a result of technical issues or due to low expression of some of these genes, impairing their detection in the assembled transcriptome. Genomic information about this species will be important to refine these results.

CYP6 is the most abundant CYP3 family in the herbivorous species analyzed (37.3% of CYP3 are CYP6 in *H. halys*, 36.5% in *N. viridula,* and 68.75% in *N. lugens*) and in *C. lectularius* (26.7%), and the second largest in *R. prolixus* (15.1%). However, our phylogenetic analysis indicates that a number of the sequences previously annotated as CYP6 actually belong to different families (Fig. 2C). Within CYP6 family, LV subfamily seems to be exclusive to pentatomids (18 in *H. halys* and 11 in *N. viridula*). The second largest family in pentatomids and *C. lectularius*, and the most abundant in *R. prolixus*, is CYP395 (20% of the sequences in *H. halys*, 12.2% in *N. viridula*, 23.3% in *C. lectularius,* and 26.4% in *R. prolixus*). However, none of the *N. lugens* CYP sequences was classified into this family. CYP395-S, CYP395-R, CYP395-P and CYP395-Q subfamilies are only represented in pentatomids, CYP395-C to CYP395-F subfamilies contain only *R. prolixus* sequences, and CYP395-A and CYP395-B subfamilies are represented only by *C. lectularius* sequences. CYP3225 to CYP3231 families are exclusive to pentatomids. CYP3225 family is larger in *H. halys* (12 sequences) when compared to *N. viridula* (3 sequences). On the contrary, CYP3226 subfamily contains 2 *H. halys* and 5 *N. viridula genes*. CYP3227 family contains 6 *H. halys* and 4 *N. viridula* sequences, whereas CYP3228 to CYP3231 families are represented by one gene in both *H. halys* and *N. viridula*.

CYP3084 to CYP3089 and CYP3091 families are absent in pentatomids. The latter is represented by 5 sequences in *R. prolixus* and 1 in *C. lectularius*. Families exclusive to *R. prolixus* are CYP3084, CYP3085, CYP3086, CYP3088, and CYP3096. CYP3090 family, which was previously described only in triatomines [4], seems to be ubiquitous in Heteroptera with 1 sequence in *H. halys*, *N. viridula, R. prolixus*, and *C. lectularius*. Finally, CYP3092 family, described for triatomines for the first time in previous works [4, 16], was also detected in pentatomids (4 sequences in *R. prolixus* and *N. viridula,* and 7 in *H. halys*). Within this family, CYP3092A subfamily is exclusive to *R. prolixus*, whereas subfamilies CYP3092D and CYP3092E were only detected in the pentatomids.

We observed that CYP9 family (belonging to CYP3 clan) was absent in all the hemipteran databases analyzed here (Supplementary Fig. 1C). In previous work, we reported the absence of CYP9 family in triatomine species [4]. Furthermore, a recent work analyzing the genome of the predaceous hemipteran *Orius laevigatus* (Anthocoridae) also reported the lack of CYP9 family [18]. Globally, results point to the lack of CYP9 family as a common observation in hemipteran genomes. Given that CYP9 is a large family in insect genomes belonging to different orders, and that it was associated with insecticide resistance and xenobiotic detoxification [11], the lack of this family in Hemiptera could be a relevant finding for evolutive and applied entomology. On the contrary, CYP395 family is one of the largest groups in heteropteran databases. Given the absence of the CYP9 family, CYP395 could have a role in detoxification in heteropterans.

#### CYP4 clan

CYP4 is the second most numerous CYP clan in the genomes of insects of different orders [17]. Many genes belonging to this clan have been involved in detoxification [19]. The analysis revealed 47, 43, 32, 12, and 34 CYP4 genes in *R. prolixus*, *H. halys*, *N. viridula, C. lectularius,* and *N. lugens*, respectively. CYP4 family was significantly larger with respect to the whole CYP complement in *N. lugens* compared to the CYP complements in *H. halys* and *C. lectularius* (Table A in Supplementary information 1). This was due to the small number of CYP3 genes detected in the planthopper. A significant reduction of CYP4 was also observed in *C. lectularius* compared to *N. viridula* and *R. prolixus* (Table 1, Fig. 2D, Table A in Supplementary information 1). This result reflects an expansion of the clan in *R. prolixus* due to 38 gene duplication events (Table 2). Our methodology cannot rule out that the expansion occurred in the ancestor of the hematophagous species with a subsequent massive gene loss in *C. lectularius*. A genus-specific expansion of CYP4 was also reported for *Acyrthosiphon* spp. compared to other Aphididae [9]; in that case, the authors proposed gene loss in other members of the subfamily, and a conservation of the ancestral numbers in *Acyrthosiphon*. A large number of duplications (27) was detected in the ancestor of the pentatomids (Table 2). Again, the losses detected in *N. viridula* should be confirmed with genomic information.

CYP4 genes were classified into CYP4, CYP3222, CYP3223, CYP3224, CYP380, CYP417, CYP425, CYP426, CYP439, and CYP3093 families, 3222 to 3224 being exclusive to pentatomids, and 380, 417, 425, 426, and 439 exclusive to *N. lugens* (Fig. 2D). Besides, CYP3093 family was exclusive and expanded in *R. prolixus* (71.4% of the CYP4 clan) due to a gene bloom (Fig. 2D). It was proposed that expansions of CYP genes occur in response to environmental stimuli, leading to a potential development of insecticide resistance [11]. To date, there is no functional information on CYP3093 for *R. prolixus*, with the exception of bioinformatic molecular docking models. For several CYP3093 members, these models proposed a favorable interaction between the pyrethroid deltamethrin and the active site, suggesting a possible role in insecticide metabolism [20]. In *T. infestans* CYP3093 is highly expressed in tegument, which is the first barrier to toxics, and is overexpressed in resistant *T. infestans* populations [20]. Altogether, the evidence allows us to hypothesize that CYP3093 expansion confers to *R. prolixus* the potential to acquire resistance to chemical insecticides. The bloom of CYP3093 observed in *R. prolixus* is not shared by other triatomine species [4], nor by other heteropterans (present results), suggesting that it may be a recent event in the evolution of this species.

A hundred percent of the CYP4 sequences of *C. lectularius* (12) belong to the CYP4 family, which is also represented in *R. prolixus* (14 genes, 29.8% of the clan), *H. halys* (30 genes, 69.8%), *N. viridula* (20 genes, 62.5%), and *N. lugens* (17 genes, 50%). However, our phylogenetic analysis suggests that there may be greater diversity within this family, as the sequences are grouped in several different clades. It has been suggested that CYP4 family plays a role in insecticide tolerance and resistance in triatomine species, given their higher expression in *T. infestans* resistant to pyrethroids [4, 21]. Moreover, RNAi mediated gene-silencing of some CYP4 family members led to an increased susceptibility to deltamethrin in *T. infestans* [22] and *R. prolixus* [23].

### Carboxyl/cholinesterases

Database searches and manual gene curation (Table B in Supplementary information 4) revealed 50 genes encoding CCEs in *N. lugens*, 72 in *H. halys*, 46 in *C. lectularius,* and 52 in *N. viridula*, whereas the *R. prolixus* genome includes 63 CCE genes (Table 1). This is a dynamic detoxification gene family with 184 duplications and 51 gene losses detected (Table 2, Fig. 1). These numbers suggest an expansion in the superfamily in Hemiptera (Table A in Supplementary information 1), which was more pronounced in some branches of the phylogeny. Interestingly, very large expansions occurred in the ancestors of pentatomids (44 duplications) and individually in *R. prolixus* (39), *C. lectularius* (24), and *H. halys* (33). Overall, this dynamic was mostly branch-specific, suggesting that it could be the result of relatively recent adaptation to different environmental niches. The number of gene losses (14) is certainly overestimated due to the lack of genomic data in *N. viridula*.

The CCE superfamily includes proteins with different functions that were classified into three functional classes [24, 25]: neuro/developmental (ND) class that, with the exception of acetylcholinesterases (AChE), lacks catalytic activity; dietary/detoxification (DD) class, and hormone and pheromone processing (HPP) esterases. In previous work, we showed that triatomine species lack DD class, and that most of the CCEs in these species were classified as HPP [4]. More recently, Bailey et al. [18] also reported the lack of DD in the predaceous heteropteran *O. laevigatus.*

We classified CCE sequences of *N. lugens*, *N. viridula,* and *C. lectularius* based on their phylogenetic relationships to previously reported CCEs from *H. halys* and *R. prolixus* [4, 13] and the analysis of characteristic conserved residues in their sequences (Supplementary information 2, Fig. 3). Interestingly, DD class was only represented in *N. lugens* (5 genes). The lack of CCE DD class in blood-feeding and predaceous Hemiptera has been proposed as a consequence of their diet; given that these species are not exposed to plant secondary metabolites, they would not need this class of CCEs [4, 18]. However, our results revealed that DD CCEs are also absent in pentatomids, suggesting its loss during the evolution of Heteroptera. This is also reinforced by evidence of DD CCEs in non-Heteroptera hemipteran species, such as Aphidinae [9].

**Fig. 3:**
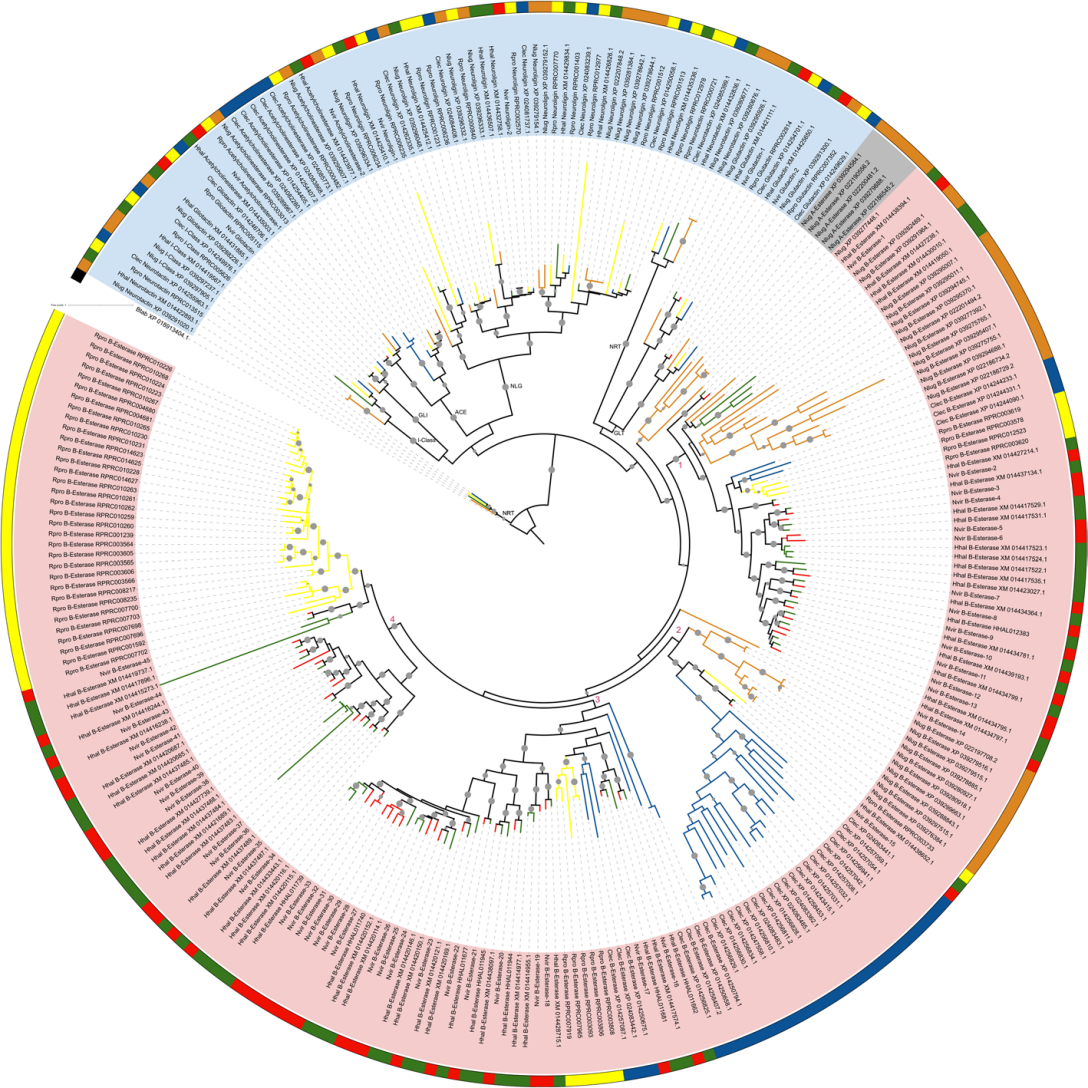
Phylogeny of CCE superfamily from *N. viridula* (Red: Nvir), *R. prolixus* (Yellow: Rpro), *H. halys* (Green: Hhal), *N. lugens* (Orange: Nlug), and *C. lectularius* (Blue: Clec). Classes are highlighted in light blue (Neurodevelopment), gray (Dietary) and pink (Hormone and pheromone processing). Cholinesterase 1 from *B. tabaci* was used as an outgroup (XP_018913404.1 - NCBI), and the tree was rooted on this sequence. Branch support values > 80 are marked to scale with a gray circle

The sequences considered as HPP were defined here taking into account phylogenetic relationships and the presence of previously annotated β-esterases [13]. In agreement with previous reports on Heteroptera [4, 18], we observed an important diversification in the HPP. Forty-three CCE HPP class were detected in *R. prolixus*, 30 in *C. lectularius*, 57 in *H. halys*, 45 in *N. viridula,* and 25 in *N. lugens*. These numbers from Heteroptera are considerably high er than the numbers reported for species of other insect orders, and even for other hemipterans [9]. One CCE sequence of *N. lugens* and 20 of *C. lectularius* could not be classified as β-esterases by sequence identity analysis, but they are part of the hormone and pheromone processing class according to the phylogeny (Fig. 3). Large expansions in HPP from heteropteran, especially pentatomids, were observed. To facilitate comparisons, the HPP groups were numbered from 1 to 4 (Fig. 3). *Drosophila melanogaster* and *N. lugens* sequences are present in groups 1 and 2 (Supplementary Fig. 2). The brown planthopper showed an expansion of HPP sequences in both groups, whereas expansions from pentatomids and *C. lectularius* were also observed in groups 1 and 2, respectively. In group 3, where only heteropteran sequences occur, the pentatomid species also presented an expansion, which is phylogenetically related to a clade containing between 2 and 5 sequences in each heteropteran species analyzed here. In group 4, *R. prolixus*, as well as the pentatomid species, presented a large expansion although they seem to be independent. Interestingly, no *C. lectularius* sequences were found in this group (Fig. 3).

The ND class encodes neuroligin, gliotactin, glutactin, and neurotactin proteins, which are not catalytic. It also encodes AChE, which is involved in neurotransmission, and is the target site of organophosphate and carbamate insecticides [26]. ND class presented smaller species-specific expansions than the HPP class in the species analyzed. In the ND class, the gene complement was significantly expanded in *R. prolixus* and *C. lectularius* with respect to *N. viridula,* and in *N. lugens* with respect to *N. viridula* and *H. halys* (Table A in Supplementary information 1). *Drosophila melanogaster* and other dipteran genomes possess 1 AChE encoding gene. A single gene encoding AChE was also found in *N. viridula* transcriptome, while *R. prolixus*, *H. halys,* and *N. lugens* had 2 each. Remarkably, the *C. lectularius* genome encodes 5 AChE paralogue genes (Fig. 3). All the species analyzed possess 1 gene classified as “putative neuroreceptor” (gliotactin or clade K) within the neurodevelopmental class. *H halys*, *R. prolixus,* and *C. lectularius* have 2 genes in the neurotactin group and 1 in the “uncharacterized” or I group, *N. lugens* has 2 genes in I group and 3 in neurotactin, whereas *N. viridula* transcriptome does not have any representatives in these groups. All species analyzed herein have 2 genes in the glutactin group. The most numerous group in the neurodevelopmental class is the one formed by neuroligins: 2 in *N. viridula*, 5 in *C. lectularius*, 7 in *H. halys*, 10 in *N. lugens,* and a significant expansion with 12 representatives in *R. prolixus* (Table A in Supplementary information 1).

Altogether, our results and previous reports suggest a particular configuration of the CCE complement in Heteroptera, with a lack of DD and expansions in the HPP class. In the absence of DD class, catalytic β-esterases could have a role in detoxification in Heteroptera. In this sense, the expansions observed in the HPP class may functionally counteract the absence of DD in the ability of these species to cope with toxic xenobiotics.

### Glutathione transferases

GST enzymes play a fundamental role in the detoxification of endogenous and xenobiotic compounds, but they also participate in hormone biosynthesis, intracellular transport, and protection against oxidative stress [27]. These enzymes can metabolize compounds by reductive dehydrochlorination or by conjugation reactions with reduced glutathione, generating soluble metabolites that are easier to eliminate. GSTs are classified as microsomal, mitochondrial and cytosolic, depending on their location in the cell; mitochondrial GSTs have not been found in insects. While 7 types of cytosolic GSTs have been recognized in mammals, insects have 4 of these classes known as omega, sigma, theta, and zeta. Delta and epsilon classes, associated with insecticide resistance in Diptera, were only reported in insects to date [27].

Fourteen GSTs were found in *R. prolixus*, 16 in *N. viridula,* 15 in *C. lectularius* and *N. lugens*, and 29 in *H. halys* (Table 1). Although this superfamily was less dynamic in terms of gene birth and death as compared to the other detoxification-related superfamilies, it also presented an expansion trend (Fig. 1). The largest number of duplications was observed in the ancestor of pentatomids, suggesting an adaptive role of this superfamily in phytophagous heteropterans. The absence of genomic data for *N. viridula* may be hiding duplications in this species and leading to an overestimation of the number of gene losses (14). The expansion of the GST superfamily observed in the *H. halys* genome is significant when compared with both *N. lugens* and *R. prolixus* (Table A in Supplementary information 1).

Microsomal GSTs have not been reported to play a role in the detoxification of xenobiotics; even though they differ structurally from cytosolic GSTs, they catalyze similar reactions [27]. We found 3 sequences in *H. halys* and *C. lectularius*, 2 in *N. lugens*, while *R. prolixus* and *N. viridula* databases encode only 1 microsomal GST.

**Fig. 4:**
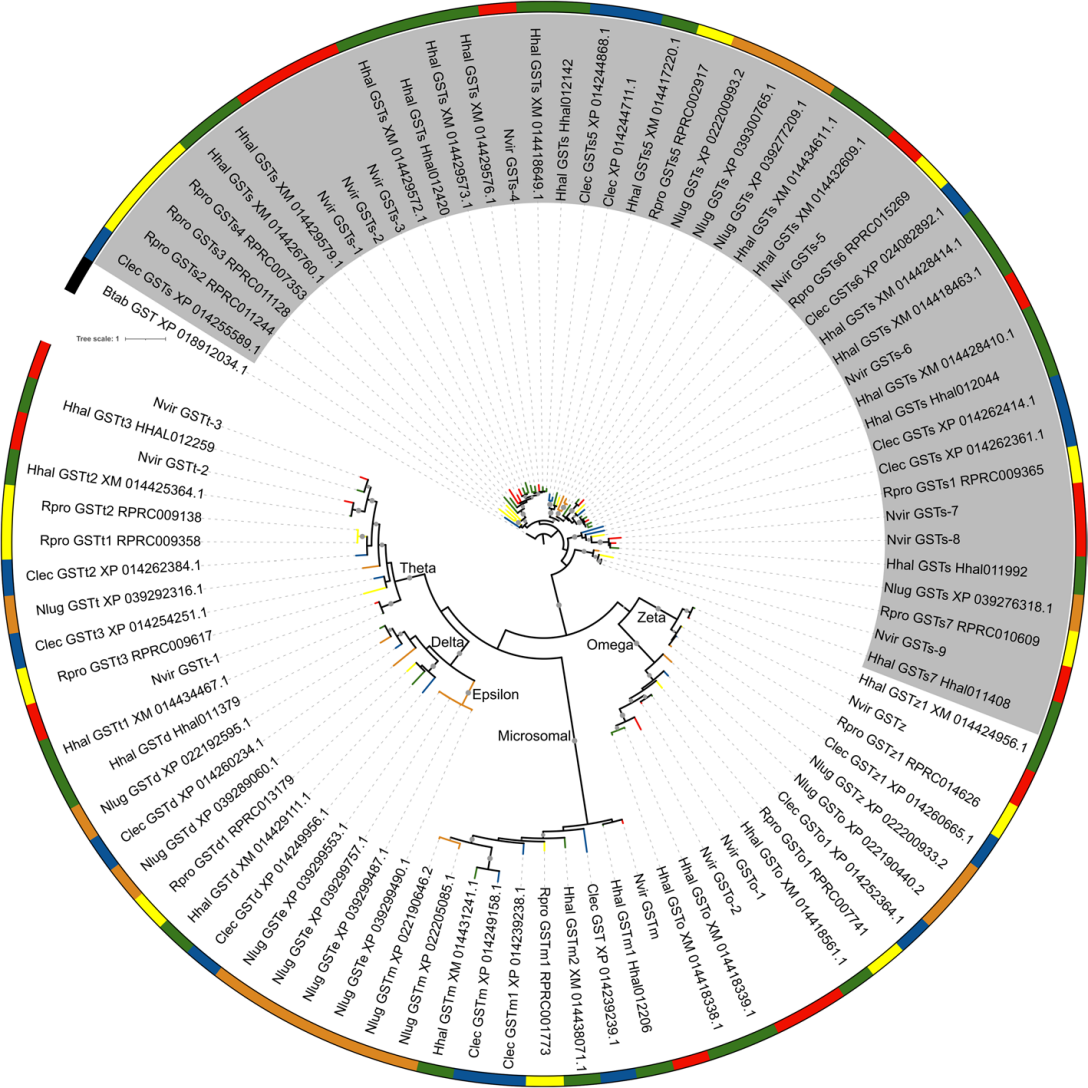
Phylogeny of GST superfamily from *N. viridula* (Red: Nvir), *R. prolixus* (Yellow: Rpro), *H. halys* (Green: Hhal), *N. lugens* (Orange: Nlug), and *C. lectularius* (Blue: Clec). The sigma class is highlighted in gray, the rest are named in the tree. A GST from *B. tabaci* was used as an outgroup (XP_018912034.1 - NCBI), and the tree was rooted on this sequence. Branch support values > 80 are marked to scale with a gray circle

The hemipteran species analyzed herein have a low number of delta (2 in *H. halys*, *N. lugens* and *C. lectularius*, 1 in *R. prolixus,* none in *N. viridula*) and epsilon GSTs (4 in *N. lugens* and none in the heteropterans, Table 1) (Fig. 4 and Supplementary Fig. 3). With the only exception of *N. lugens* and *Trialeurodes vaporariorum* [18], the absence of epsilon GSTs is a common finding in hemipteran genomes [9]. Conversely, other GST groups are larger in these species than in other insect orders [4]. Particularly, sigma class was the largest group, especially in pentatomids (Table 1). Fifty percent of the total GSTs of *R. prolixus* (7 out of 14), 56.25% in *N. viridula* (9 out of 16), 40% in *C. lectularius* (6 out of 15), 58.6% in *H. halys* (17 out of 29), and 26.7% in *N. lugens* (4 out of 15) belong to sigma class (Table 1). These results suggest that the GST sigma class could be relevant for detoxification in Hemiptera, compensating for the absence or reduction in other GST groups. However, this tendency of an expanded sigma class was not confirmed in Aphidinae; instead, delta class presented a genus-specific expansion in the genus *Acyrthosiphon* [9].

The omega class has a cysteine residue in its active site, which allows the catalysis of thiol transferase and reduction reactions that are not catalyzed by the other GST classes [28]. Among the hemipterans studied herein, *H. halys* possesses 3 genes in this class, *N. viridula* has 2, whereas *N. lugens*, *R. prolixus,* and *C. lectularius* have 1 representative each (Fig. 4, Table 1).

The theta class has been proposed to be a contributor to the detoxification of xenobiotics due to its protective action against oxidants and dehalogenation activity [6]. *R prolixus, H. halys*, and *N. viridula* have 3 genes in this class, whereas *C. lectularius* has 2, and the *N. lugens* genome encodes 1. Finally, 1 gene belonging to the zeta class was detected in each of the hemipteran databases analyzed here.

### Genomic clusters and evolution of detoxification gene superfamilies

**Fig. 5:**
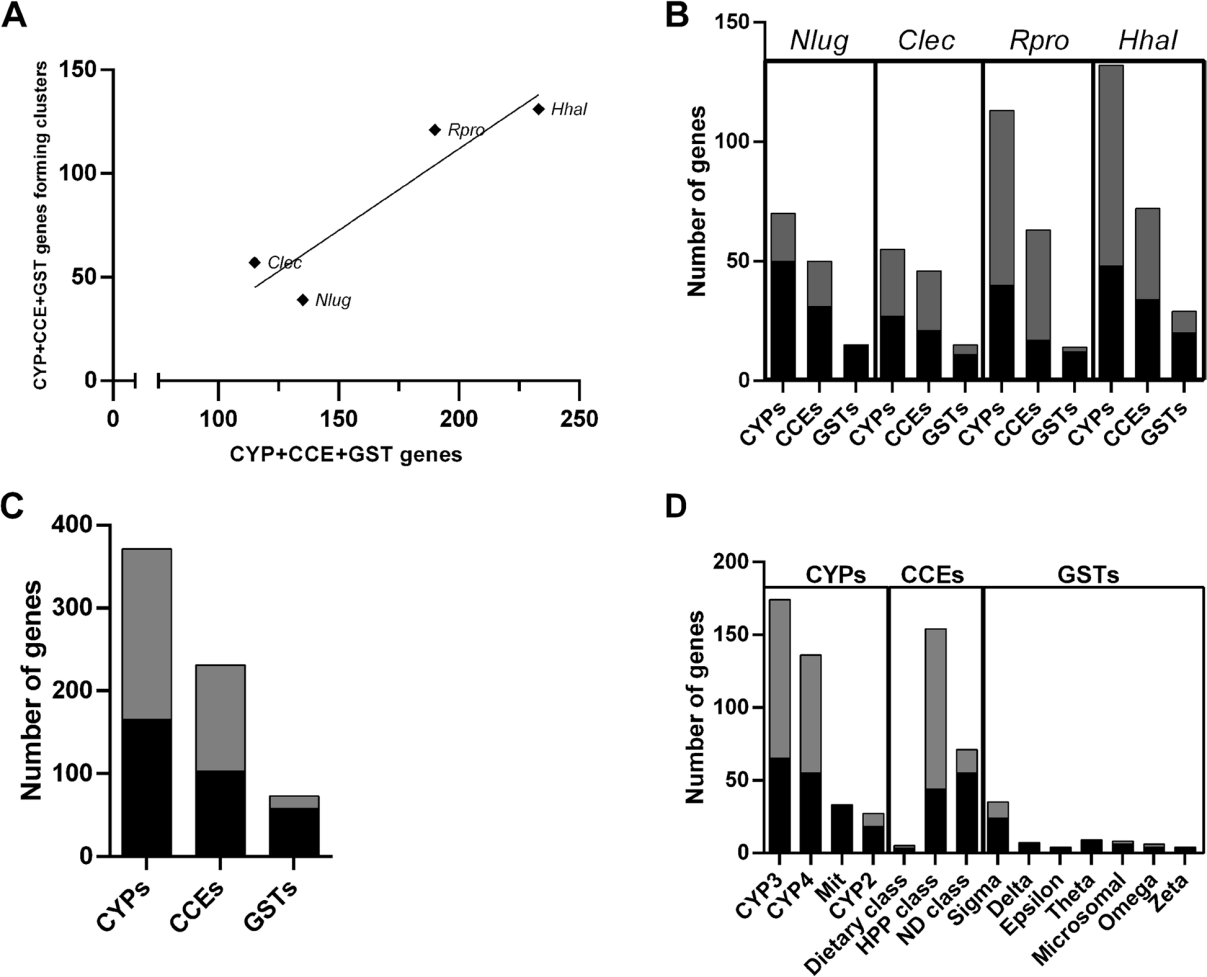
Cluster analysis across species and superfamilies. **A** Total detoxification genes vs. detoxification genes in clusters for each species with available genome. **B** Total detoxification genes vs genes in clusters in *N. lugens* (Nlug), *C. lectularius* (Clec), *R. prolixus* (Rpro) and *H. halys* (Hhal) for each superfamily under analysis. **C** Sum of total genes vs. sum of genes in clusters for each superfamily under analysis. **D** Total genes vs. genes in clusters for each clan/class under analysis. Black bar: number of genes outside gene-clusters; Gray bar: number of clustered genes

Paralogue genes that originated from relatively recent duplications are usually organized in clusters in the genomes. Furthermore, duplicated genes that are in adjacent genomic regions could be regulated in a coordinated way. For this reason, it is possible that evolutive forces to maintain genes in a cluster organization, even a long time after the duplication events [29]. Accordingly, a positive linear correlation was observed between the total number of detoxification genes (CYP, CCE, and GST families) and the detoxification genes organized in genomic clusters (*R* = 0.96) (Fig. 5A). This tendency is maintained when each superfamily is considered separately (*R* = 0.99 for GSTs; *R* = 0.99 for CYPs; *R* = 0.81 for CCEs). The detoxification genes forming clusters represented 28.9% in *N. lugens*, 49.13% in *C. lectularius*, 63.7% in *R. prolixus,* and 56.22% in *H. halys* (Fig. 5B).

Considering the total of CYP members identified in the four species under analysis with available genomic sequence, 54% form clusters; these percentages are 55.6 for CCEs and 20.5 for GSTs (Fig. 5C). All families within the CYP superfamily showed a tendency to form clusters, except for the mitochondrial clan. CYP2 contains 33.3% of genes in clusters, CYP3 62.6%, and CYP4 59.6% (Fig. 5D). The large percentage of genes in clusters is in accordance with a large number of species-specific duplications observed in the CYP3 and CYP4 gene families, suggesting recent duplication events.

**Fig. 6:**
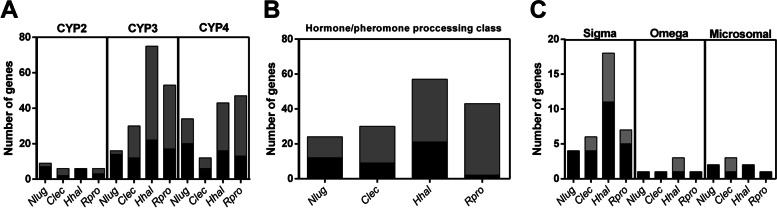
Families with the majority of their genes encoded in gene clusters were analyzed on a species-by-species basis. **A** CYP. **B** CCE. **C** GST. Black bar: number of genes outside gene-clusters; Gray bar: number of clustered genes

Most of the CYP3 and CYP4 genes were clustered in the heteropterans (Fig. 6A); whereas most of the genes belonging to CYP2 were clustered in the hematophagous species analyzed. HPP class of CCE has 71.4% of genes located in clusters (Fig. 6B). This tendency is strong in Heteroptera, being evident in *R. prolixus* (95% of clustered genes) (Fig. 6B). In the case of the GSTs, the highest proportion of clustered genes (close to 30%) is given by the sigma, microsomal, and omega classes (Figs. 5D and 6C).

Our analysis of gene gains and losses revealed some interesting patterns. The large number of both gene duplications and losses suggests that the detoxification gene superfamilies have a very dynamic evolution, with high turnover rates resulting in many species and lineage-specific genes. This evolutionary pattern resembles that of chemosensory gene families that similarly to the detoxification gene families, have a large adaptive potential [29]. Interestingly, it has been recently suggested that some chemosensory genes are involved in detoxification in insects, probably by sequestering toxic molecules [30–32].

Among the detoxification superfamilies, the CYP superfamily was the most dynamic with a total of 354 events (duplication and losses), the majority of which were duplications, indicating that this family is expanding. The CYP3 and CYP4 clans were the most dynamic within the superfamily (Fig. 1, Table 2). The second more dynamic superfamily was CCEs, with 235 events. The GST superfamily had fewer events in comparison, which is in accordance with having less genes than the other superfamilies. Overall, all families had more duplications than losses, which suggests a general expansion trend.

Comparisons across species showed an expansion of detoxification-related genes in the lineage, leading to the phytophagous heteropterans (Fig. 1). This is especially true for CCE and CYP superfamilies. The GST superfamily had fewer events overall, but also appears to be more dynamic in the phytophagous as compared to the hematophagous species. Besides, a large expansion was observed in CYP in *H. halys*, which also had many duplications in the other superfamilies. The high number of duplications in phytophagous lineages is in accordance with phytophagy being a newly acquired diet in the group from the predator common ancestor of all the heteropterans [1]. A phytophagous diet represents an important adaptive challenge, as a result of the arms race between plants trying to avoid herbivory and insects trying to overcome the toxicity of chemical compounds produced by plants. The broad complement of detoxification enzymes resulting from both ancestral and species-specific duplication in *H. halys* is certainly instrumental in its feeding on different plant species, facilitating its success as an invasive species. *Nezara viridula* had a very different pattern, most likely as a consequence of having only transcriptomic data available for this analysis. Due to the incompleteness of transcriptomes, gene numbers and duplications are probably underestimated, while gene losses are likely overestimated in this species. However, *H. halys* is a polyphagous pentatomid, suggesting that the observation of a broader complement of detoxification enzymes in comparison with *N. viridula*, may not be a consequence of the lack of genomic information in the latter, but could be related to different feeding habits between the species.

In contrast with the phytophagous species, significant expansions were not observed in the hematophagous ancestors, which in turn had many species-specific expansions. The GST superfamily, however, seems to be shrinking among the blood-feeding heteropterans. Hematophagy evolved independently in *C. lectularius* and *R. prolixus* as their most recent common ancestor was likely a predator [1]. Hence, it makes sense that these two species had independent evolution of their detoxification gene complements in response to the diet change, with many species-specific duplications and losses. *Rhodnius prolixus*, however, had many more duplications than *C. lectularius*, which accounts for most of the large difference in the total number of detoxification genes between these two species. In particular, there was a noticeable difference between the two hematophagous species in the evolutionary dynamics of CYP4 family, in which *R. prolixus* had 38 gene duplications, while *C. lectularius* had none.

### Concluding remarks

Here we present a comparative analysis of detoxification gene superfamilies among heteropteran species with different feeding specializations. The availability of transcriptomic data allowed us to include *N. viridula*, an important crop pest that is still understudied. Nevertheless, a complete genomic sequence of the species is essential to close gaps in the understanding of its detoxification gene complement and in the evolution of these genes in Heteroptera. Our results indicate a reduction in several enzyme families associated with xenobiotic detoxification in the four heteropteran species analyzed here: absence of the epsilon class and a reduced delta class in the GST superfamily; absence of the mitochondrial CYP12 family; absence of the CYP9 family in CYP3 clan; and absence of the DD class of CCEs. Conversely, other detoxification-related families are expanded in the group, and have had a dynamic evolution: CYP3 clan, HPP in the CCE superfamily, and sigma class in GST superfamily. These characteristics were previously proposed as particularities of the Triatomine subfamily, or even for non-herbivorous heteropterans [4, 18]. Our results demonstrate that these features could be extended to the Heteroptera suborder.

Our comparative analysis suggests that diet may be an important driver of detoxification gene family evolution in Heteroptera. Nevertheless, a sampling of more species would be necessary to check this hypothesis. For instance, it would be interesting to include more species belonging to Cimicomorpha, both phytophagous and carnivorous, to separate diet from phylogenetic constraints in the evolution of the detoxification gene superfamilies in this group. In addition, the inclusion of non-pentatomid phytophagous species would allow checking whether switching to an herbivorous diet is associated with an increase in the detoxification gene repertoire. On the other hand, it would be interesting to study the particular genes involved in the parallel evolution of blood compound detoxification in *C. lectularius* and *R. prolixus*. Finally, given that detoxification in insects is revealing to be an extremely complex trait, which involves multiple gene families including ABC transporters, UDP-glycosyltransferases, transcription factors, heat shock proteins, chemosensory proteins, cuticular proteins, and even changes in the microbiome (see, for example, [30–34]), a comparative analysis of additional gene families will give a more complete panorama of detoxification potential in different species.

## Methods

### Gene identification and sequence analysis

BLASTp searches (with expectation value threshold < 0.0001) were performed using the PFAM domains PF02798 and PF00043 (GSTs), PF00135 (CCEs), and PF00067 (CYPs) as queries in the predicted protein datasets of *C. lectularius* (NCBI accession number GCF_000648675.2), *H. halys* (i5k number OGS1.2), *R. prolixus* (NCBI accession number GCA_000181055.3), and *N. lugens* (NCBI accession number GCA_014356525.1). The PFAM domains mentioned above were used as queries to perform tBLASTn searches [35] on a *N. viridula* nonredundant transcriptome [12]. All the positive hits against the transcriptome were manually curated and analyzed using homologous sequences as reference to reconstruct transcripts whenever evident errors in sequencing (such as frameshifts or misassemblies) were detected. Transcripts encoding proteins shorter than 100 amino acids were discarded. The longest isoform from each of the resulting genes from BLASTp and the sequences obtained from tBLASTn searches were used as queries to perform new searches on the mentioned databases. BLASTp and tBLASTn searches were performed on the genome predicted proteins and on *N. viridula* transcriptome, respectively, with an E-value threshold < 0.0001. Protein sequences shorter than 100 amino acids were discarded.

The predicted proteins obtained from the genomes under study were analyzed to keep only the longest isoform from each gene for subsequent analyses. Positive hits resulting from all the searches were re analyzed in two sequential steps: an InterProScan search [36] using the Gene3d, PfamA, and SuperFamily applications, and BLASTp searches against the nonredundant database on the NCBI. Those hits not belonging to the superfamilies of interest were discarded. Sequences encoding CYP s, CCEs, and GSTs from the *N. viridula* transcriptome are presented in Supplementary information 3*.* Accession numbers of all sequences used for the analysis from *N. lugens*, *C. lectularius*, *R. prolixus,* and *H. halys* are presented in Supplementary information 4.

### Phylogenetic analysis

Protein sequence alignments for the target protein families were generated with MAFFT [37] using the G-INS-i option with the following settings: “leave gappy regions” active; Unaligned level = 0.1; Offset value = 0.12; Max iterate = 1000. The alignments were trimmed using trimAl v1.2 [38] with default parameters except for the gap threshold (−gt) that was fixed at 0.3. G-INS-i is slower but more accurate than other methods (for details see [37]). The trimmed alignments were used to build a phylogenetic tree for each family based on the approximately maximum likelihood approach with IQ-tree v 1.6.12 [39] using -B 1000 and -alrt 1000 settings to combine ModelFinder option, tree searching based on 1000 replicates, and estimation of branch support using the approximate Likelihood Ratio Test based on the Shimodaira-Hasegawa (aLRT-SH) procedure [40]. Branch support values between 80 and 100 were marked with a circle to scale. The best-fit amino acid substitution model estimated by IQ-tree for each protein family and chosen according to Bayesian Information Criterion (BIC) was: LG + F + R4 in Mitochondrial CYP clan (Fig. 2A), LG + I + G4 for CYP2 (Fig. 2B), Q.yeast+R7 for CYP3 (Fig. 2C), Q.pfam+F + R6 for CYP4 (Fig. 2D), WAG+F + R8 for CCEs (Fig. 3), and LG + R4 for GSTs (Fig. 4). The best-fit amino acid substitution model for each family was estimated by ModelFinder, integrated on IQ-tree software. It selects the best model among hundreds of options using the Bayesian Information Criterion [41]. The phylogenetic trees were outgroup-rooted and edited using iTol online tool (https://itol.embl.de).

The sequences were named according to previous annotations available for *R. prolixus* [4, 16], *H. halys* [13], *C. lectularius* [42], and *N. lugens* [43]. These annotations, along with the phylogenetic relationships observed in the trees, were also used to name the new sequences found in our analysis. Additionally, for the classification of sequences into clans, families, and subfamilies, we included *D. melanogaster* as reference in additional phylogenetic analyses, as it represents a model organism with a well-studied genome. The trees were constructed following the same methodology described above, using the following substitution models: LG + F + R5 (Mitochondrial CYP clan, Supplementary Fig. 1A), LG + I + G4 (CYP2 clan, Supplementary Fig. 1B), Q.yeast+R7 (CYP3 clan, Supplementary Fig. 1C), Q.pfam+F + R8 (CYP4 clan, Supplementary Fig. 1D), WAG+F + R9 (CCEs, Supplementary Fig. 2) and Q.pfam+R6 (GSTs, Supplementary Fig. 3). Once phylogenetic trees were generated, sequence identity analyses were performed using CD-HIT with an identity threshold of 30% to confirm the proposed annotation. CCE sequences were aligned to a reference from *D. melanogaster* (CG17907) using Clustal Omega [44], and the presence of conserved regions and specific residues along the sequences was verified to confirm their classification (Supplementary information 2).

### Duplication analysis and enrichment statistical analysis

Chi-square tests were performed in order to identify significant expansions in gene families. Two-by-two contingency tables (Tables B-E in Supplementary information 1) were constructed between two species and:

- The number of sequences encoding either CYPs, CCEs or GSTs vs. the rest of genes of a particular genome.
- The number of sequences encoding a particular detoxification superfamily (CYPs, CCEs or GSTs) vs. the sum of genes belonging to the other two superfamilies under study.
- The number of sequences encoding enzymes from a particular group belonging to CYPs, CCEs or GSTs vs. the rest of sequences of the same superfamily.

To estimate the number of gene duplications and losses, we used the gene tree vs. species tree reconciliation method. This method compares gene trees with the species tree and identifies gene gains and losses that could explain the differences between them. In this way, we manually inspected the phylogenetic trees obtained for each family following [45]. This method may underestimate the number of events, as it does not take into account genes that may have been gained and were then lost (we did not analyze pseudogenes). However, because each orthogroup is analyzed separately, the results are more accurate than when automated methods are applied [45]. The objective was to have an overall idea of the gene birth-and-death dynamics of each family and detect differences between species and clans/classes. It is important to bear in mind that the estimates of gene gain and losses are dependent on the annotations; faulty annotations will lead to an overestimation of losses and underestimation of gains.

### Cluster analysis

General feature format (GFF) files were downloaded for *R. prolixus* (GCA_000181055.3_Rhodnius_prolixus-3.0.3.gff) from NCBI; *H. halys* (halhal_OGSv1.2.gff) from i5K; *C. lectularius* (GCF_000648675.2_Clec_2.1_genomic.gff) from NCBI; and *N. lugens* (GCF_014356525.1.gff) from NCBI. Target gene locations (coordinates and scaffold) for the four species were obtained from each GFF file using a list of IDs and the Seqtk tool. Two genes were considered in a genomic cluster when they belonged to the same gene family and were separated by less than 35,000 bp. *Rhodnius prolixus*, *C. lectularius*, and *H. halys* genomes are assembled at the scaffold level (www.vectorbase.org; http://i5k.github.io/genomes). Hence, this should be considered as a limitation that could lead to underestimation of the total number of clustered genes, given that two genes could be close but located in different scaffolds, impairing their identification as part of a cluster. The complete reconstruction of the genomes at chromosome level of all these species will make it possible in the future to adjust cluster definition. The results presented here will be useful to pose hypotheses regarding duplication events and possible coordinated gene regulation.

## Supplementary Information

**Additional file 1: Supplementary information 1.** Table A. Comparison between superfamilies, families, and total detoxification genes by Chi-square test on the analyzed species. Table B. Two-by-two contingency table of Chi-square tests on detoxification-related genes vs. the total number of genes from each species. Table C. Two-by-two contingency table of Chi-square tests on CYP superfamily/clans vs. the total number of genes belonging to other detoxification-related superfamilies/clans. Table D. Two-by-two contingency table of Chi-square tests on CCE superfamily/class/family vs. the total number of genes belonging to other detoxification-related superfamilies/classes. Table E. Two-by-two contingency table of Chi-square tests on GST superfamily/class vs. the total number of genes belonging to other detoxification-related superfamilies/classes.

**Additional file 2: Supplementary Figure 1.** Phylogeny of CYP superfamily from *N. viridula* (Red: Nvir), *R. prolixus* (Yellow: Rpro), *H. halys* (Green: Hhal), *C. lectularius* (Blue: Clec), *D. melanogaster* (Purple: Dmel), and *N. lugens* (Orange: Nlug). A) Mitochondrial clan. B) CYP2 clan. C) CYP3 clan. D) CYP4 clan. A CYP gene from *Bemisia tabaci* was used as an outgroup (AEK21835.1 - NCBI), and the tree was rooted on this sequence. Branch support values > 80 are marked to scale with a gray circle.

**Additional file 3: Supplementary information 4.** Protein and gene accession numbers and tree’s ID for each sequence used in this study. Table A. CYP superfamily. Table B. CCE superfamily. Table C. GST superfamily.

**Additional file 4: Supplementary information 2.** Analysis of characteristic conserved residues in CCE sequences compared with Ace (CG17907) from *D. melanogaster*.

**Additional file 5: Supplementary Figure 2.** Phylogeny of CCE superfamily from *N. viridula* (Red: Nvir), *R. prolixus* (Yellow: Rpro), *H. Halys* (Green: Hhal), *N. lugens* (Orange: Nlug), *D. melanogaster* (Purple: Dmel), and *C. lectularius* (Blue: Clec). Classes are highlighted in light blue (Neurodevelopment), gray (Dietary), and pink (Hormone and pheromone processing). Cholinesterase 1 from *B. tabaci* was used as an outgroup (XP_018913404.1 - NCBI), and the tree was rooted on this sequence. Branch support values > 80 are marked to scale with a gray circle.

**Additional file 6: Supplementary Figure 3.** Phylogeny of GST superfamily from *N. viridula* (Red: Nvir), *R. prolixus* (Yellow: Rpro), *H. Halys* (Green: Hhal), *N. lugens* (Orange: Nlug), *D. melanogaster* (Purple: Dmel), and *C. lectularius* (Blue: Clec). The sigma class is highlighted in gray, the rest are named in the tree. A GST from *B. tabaci* was used as an outgroup (XP_018912034.1 - NCBI), and the tree was rooted on this sequence. Branch support values > 80 are marked to cale with a gray circle.

**Additional file 7: Supplementary information 3.** Transcript sequences in Nezara viridula database.

**Additional file 8: Supplementary information 5.** Gene-cluster analysis from each superfamily/clan for the different species analyzed in this study. Table A. *Rhodnius prolixus* genome. Table B. *Halyomorpha halys* genome. Table C. *Cimex lectularius* genome. Table D. *Nilaparvata lugens* genome.

**Additional file 9: Supplementary information 6.** Table A. Chi-square test results from the comparison of the number of genes of GSTs, CYPs and CCEs superfamilies from *C. lectularius*, *H. halys*, *R. prolixus* and *D. melanogaster* genomes and *N. viridula* transcriptome. Table B. Two-by-two contingency table of Chi-square tests on detoxification-related genes vs. the total number of genes from each analyzed species and *D. melanogaster*. Table C. Two-by-two contingency table of Chi-square tests on CYP superfamily/clans vs. the total number of genes belonging to other detoxification-related superfamilies/clans in the analyzed species and *D. melanogaster*. Table D. Two-by-two contingency table of Chi-square tests on CCE superfamily/class/family vs. the total number of genes belonging to other detoxification-related superfamilies/classes in the analyzed species and *D. melanogaster*. Table E. Two-by-two contingency table of Chi-square tests on GST superfamily/class vs. the total number of genes belonging to other detoxification-related superfamilies/classes, in the analyzed species and *D. melanogaster*.

## Data Availability

Databases used for this study are deposited under the following accession numbers: *C. lectularius:* NCBI accession number GCF_000648675.2 (https://www.ncbi.nlm.nih.gov/data-hub/genome/GCF_000648675.2/); *H. halys:* i5k number OGS1.2 (https://i5k.nal.usda.gov/bio_data/836785); *R. prolixus:* NCBI accession number GCA_000181055.3 (https://www.ncbi.nlm.nih.gov/data-hub/genome/GCA_000181055.3/); *N. lugens*: NCBI accession number GCA_014356525.1 (https://www.ncbi.nlm.nih.gov/data-hub/genome/GCF_014356525.1/). The assembled transcriptomic dataset for *N. viridula* is available at the NCBI-TSA GGPJ00000000 (https://www.ncbi.nlm.nih.gov/nuccore/1551470193).

## Abbreviations

ABC transporters: ATP-binding cassette transporters
AChE: Acetylcholinesterase
aLRT-SH: Approximate Likelihood Ratio Test based on the Shimodaira-Hasegawa procedure
BIC: Bayesian Information Criterion
BLAST: Basic Local Alignment Search Tool
CCEs: Carboxyl/cholinesterases
CD-HIT: Cluster Database at High Identity with Tolerance
CYPs: Cytochromes P450
DD: Dietary/Detoxification class
GFF: General Feature Format
GSTs: Glutathione transferases
HPP: Hormone and pheromone processing class
IDs: Identification
iTOL: Interactive Tree Of Life
i5k: Sequencing Five Thousand Arthropod Genomes
MAFFT: Multiple Alignment using Fast Fourier Transform
NCBI: National Center for Biotechnology Information
ND: Neurodevelopmental class
trimAl: A tool for automated Alignment trimming
UFBoot: UltraFast bootstrap
