# Supplementary material for: Comparative analysis of detoxification-related gene superfamilies across five hemipteran species
Source: BMC Genomics. 2022 Nov 17;23:757. doi: 10.1186/s12864-022-08974-y (PMC9670383; doi:10.1186/s12864-022-08974-y)

**Supplementary Figure 1.** Phylogeny of CYP superfamily from *N. viridula* (Red: Nvir), *R. prolixus* (Yellow: Rpro), *H. halys* (Green: Hhal), *C. lectularius* (Blue: Clec), *D. melanogaster* (Purple: Dmel), and *N. lugens* (Orange: Nlug). **A)** Mitochondrial clan. **B)** CYP2 clan. **C)** CYP3 clan. **D)** CYP4 clan. A CYP gene from *Bemisia tabaci* was used as an outgroup (AEK21835.1 - NCBI), and the tree was rooted on this sequence. Branch support values > 80 are marked to scale with a gray circle.


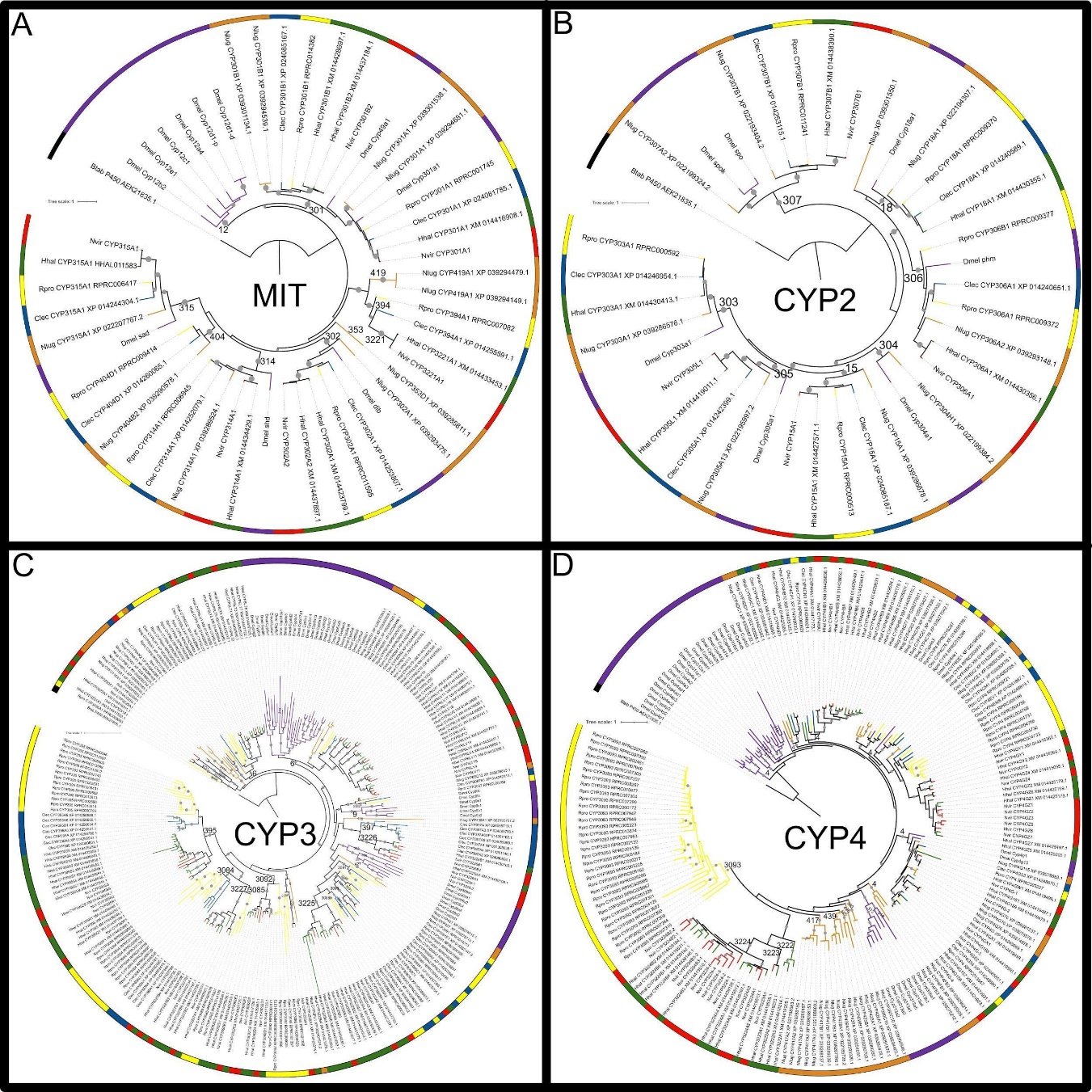

Supplement: Supplementary file 2 — Additional file 2: Supplementary Figure 1. Phylogeny of CYP superfamily from N. viridula (Red: Nvir), R. prolixus (Yellow: Rpro), H. halys (Green: Hhal), C. lectularius (Blue: Clec), D. melanogaster (Purple: Dmel), and N. lugens (Orange: Nlug). A) Mitochondrial clan. B) CYP2 clan. C) CYP3 clan. D) CYP4 clan. A CYP gene from Bemisia tabaci was used as an outgroup (AEK21835.1 - NCBI), and the tree was rooted on this sequence. Branch support values > 80 are marked to scale with a gray circle. [file 12864_2022_8974_MOESM2_ESM.docx]
