# Supplementary material for: Comparative analysis of detoxification-related gene superfamilies across five hemipteran species
Source: BMC Genomics. 2022 Nov 17;23:757. doi: 10.1186/s12864-022-08974-y (PMC9670383; doi:10.1186/s12864-022-08974-y)

**Supplementary Figure 2.** Phylogeny of CCE superfamily from *N. viridula* (Red: Nvir), *R. prolixus* (Yellow: Rpro), *H. Halys* (Green: Hhal), *N. lugens* (Orange: Nlug), *D. melanogaster* (Purple: Dmel), and *C. lectularius* (Blue: Clec). Classes are highlighted in light blue (Neurodevelopment), gray (Dietary), and pink (Hormone and pheromone processing). Cholinesterase 1 from *B. tabaci* was used as an outgroup (XP_018913404.1 - NCBI), and the tree was rooted on this sequence. Branch support values > 80 are marked to scale with a gray circle.


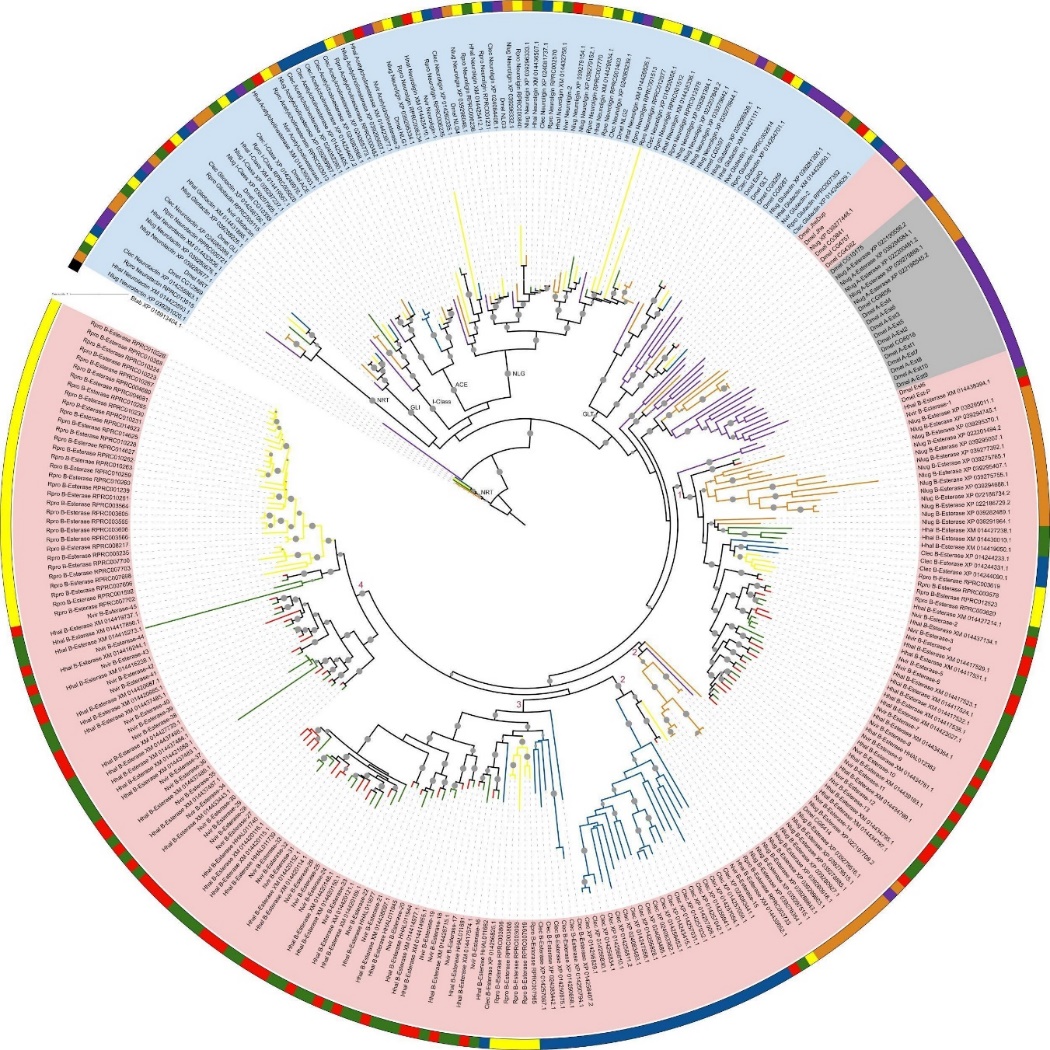

Supplement: Supplementary file 5 — Additional file 5: Supplementary Figure 2. Phylogeny of CCE superfamily from N. viridula (Red: Nvir), R. prolixus (Yellow: Rpro), H. Halys (Green: Hhal), N. lugens (Orange: Nlug), D. melanogaster (Purple: Dmel), and C. lectularius (Blue: Clec). Classes are highlighted in light blue (Neurodevelopment), gray (Dietary), and pink (Hormone and pheromone processing). Cholinesterase 1 from B. tabaci was used as an outgroup (XP_018913404.1 - NCBI), and the tree was rooted on this sequence. Branch support values > 80 are marked to scale with a gray circle. [file 12864_2022_8974_MOESM5_ESM.docx]
