# Supplementary material for: Comparative analysis of detoxification-related gene superfamilies across five hemipteran species
Source: BMC Genomics. 2022 Nov 17;23:757. doi: 10.1186/s12864-022-08974-y (PMC9670383; doi:10.1186/s12864-022-08974-y)

**Supplementary Figure 3.** Phylogeny of GST superfamily from *N. viridula* (Red: Nvir), *R. prolixus* (Yellow: Rpro), *H. Halys* (Green: Hhal), *N. lugens* (Orange: Nlug), *D. melanogaster* (Purple: Dmel), and *C. lectularius* (Blue: Clec). The sigma class is highlighted in gray, the rest are named in the tree. A GST from *B. tabaci* was used as an outgroup (XP_018912034.1 - NCBI), and the tree was rooted on this sequence. Branch support values > 80 are marked to cale with a gray circle.
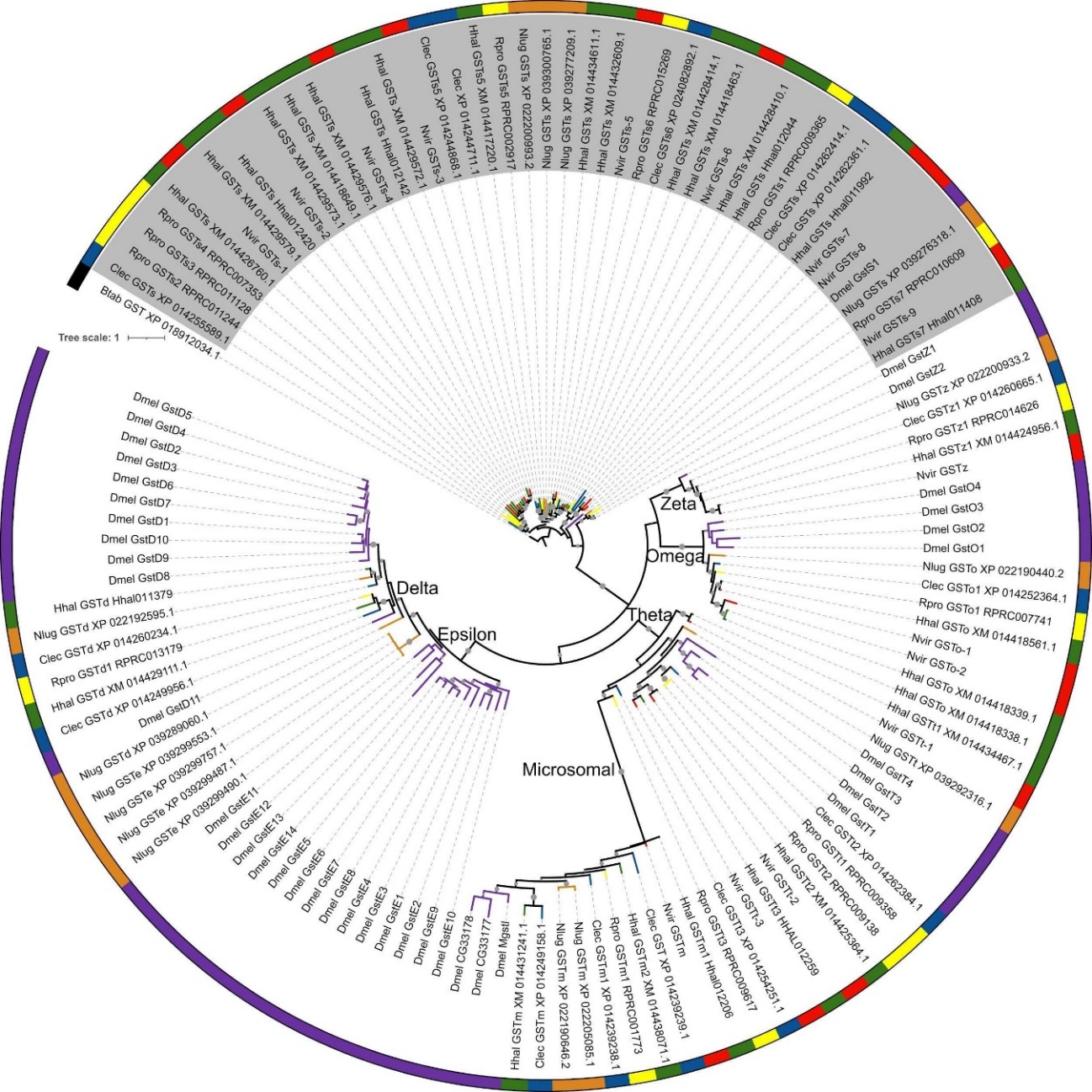

Supplement: Supplementary file 6 — Additional file 6: Supplementary Figure 3. Phylogeny of GST superfamily from N. viridula (Red: Nvir), R. prolixus (Yellow: Rpro), H. Halys (Green: Hhal), N. lugens (Orange: Nlug), D. melanogaster (Purple: Dmel), and C. lectularius (Blue: Clec). The sigma class is highlighted in gray, the rest are named in the tree. A GST from B. tabaci was used as an outgroup (XP_018912034.1 - NCBI), and the tree was rooted on this sequence. Branch support values > 80 are marked to cale with a gray circle. [file 12864_2022_8974_MOESM6_ESM.docx]
